# Effect of low complexity regions within the PvMSP3α block II on the tertiary structure of the protein and implications to immune escape mechanisms

**DOI:** 10.1186/s12900-019-0104-0

**Published:** 2019-03-27

**Authors:** Alebachew Messele Kebede, Fitsum Girma Tadesse, Adey Desta Feleke, Lemu Golassa, Endalamaw Gadisa

**Affiliations:** 1 Institute of Biotechnology, Addis Ababa University, Addis Ababa, Ethiopia; 2 Aklilu Lemma Institute of Pathobiology, Addis Ababa University, Addis Ababa, Ethiopia; 3 Radboud Institute for Health Sciences, Radboud University Medical Centre, Nijmegen, The Netherlands; 4 Armauer Hansen Research Institute (AHRI), Addis Ababa, Ethiopia

**Keywords:** Low complexity regions, Antigenic diversity, Immune evasion, Vaccine design

## Abstract

**Background:**

*Plasmodium vivax* merozoite surface protein 3α (PvMSP3α) is a promising vaccine candidate which has shown strong association with immunogenicity and protectiveness. Its use is however complicated by evolutionary plasticity features which enhance immune evasion. Low complexity regions (LCRs) provide plasticity in surface proteins of *Plasmodium* species, but its implication in vaccine design remain unexplored. Here population genetic, comparative phylogenetic and structural biology analysis was performed on the gene encoding *PvMSP3α*.

**Results:**

Three LCRs were found in *PvMSP3α* block II. Both the predicted tertiary structure of the protein and the phylogenetic trees based on this region were influenced by the presence of the LCRs. The LCRs were mainly B cell epitopes within or adjacent. In addition a repeat motif mimicking one of the B cell epitopes was found within the *PvMSP3a* block II low complexity region. This particular B cell epitope also featured rampant alanine substitutions which might impair antibody binding.

**Conclusion:**

The findings indicate that *PvMSP3α* block II possesses LCRs which might confer a strong phenotypic plasticity. The phenomenon of phenotypic plasticity and implication of LCRs in malaria immunology in general and vaccine candidate genes in particular merits further exploration.

**Electronic supplementary material:**

The online version of this article (10.1186/s12900-019-0104-0) contains supplementary material, which is available to authorized users.

## Background

In the past genetic diversity studies have been carried out to assess the circulating malaria parasite populations to assist in the formulation of strategies for monitoring and control interventions. Given that high diversity was observed in malaria and specifically in *P. vivax*, it was also important to identify the relevant polymorphisms that contribute to antigenic escape and its potential to develop a “vaccine resistant strain” [1]. To that end, a number of studies have targeted antigenic proteins using diversity covering approaches [2]. On the other hand, population genetic studies can guide vaccine design by predicting polymorphisms that contribute to antigenic diversity [3]. For *P. vivax* especially, population genetic studies are of paramount importance since it invades only reticulocytes and is notoriously hard to grow continuously in in-vitro cultures [4]. This is mainly achieved by recognizing polymorphic regions, and analyzing if the regions are under balancing or immune selection. This approach has led to substantial improvement in terms of understanding how diversity plays a role in immune escape mechanisms, but translation into current clinical trials has been hampered due to how little mechanisms underlying diversity have been studied. Most studies have so far focused on point mutations and recombination overseeing other mechanisms such as the generation of low complexity regions (LCRs) or structural adaptation of the proteins [5]. For Plasmodium species,this is perhaps most curious since LCRs are associated with host pathogen interaction and phenotypic plasticity, enhancing their role in evading the immune system [6, 7]. This is in addition to the fact that LCRs are highly frequent in Plasmodium parasite antigens.

PvMSP3α is a promising vaccine candidate with studies showing a strong association with immunogenicity and protectiveness [8]. However due to its direct contact with the immune system it is highly polymorphic with clusters of repetitive regions [1]. Repetitive regions and certain traits of phenotypic variation have been associated with low complexity regions (LCRs). LCRs contain short segments of homo-polymeric repeats, or segments that are over represented by a small number of residues of aperiodic repeats which appear as a mosaic of repeats [7]. LCRs mediate protein-protein interaction, and are involved in host pathogen communication, and immune evasion [9–13] . The influence of LCRs in MSP3α is apparent; with implications in the genetic diversity [14], the secondary structure of alpha (α) helices, its coiled-coil tertiary structure and recombination hotspots [15, 16]. Particularly extensive recombination has been largely been ascribed as the major reason behind the failure to link specific alleles to geographical regions [17–19].

In essence, studying the molecular evolution responsible for variations in *PvMSP3α*, by analyzing features such as LCRs with respect to their role in B cell epitope variability and geographical distribution is of utmost importance to guide rational vaccine design for *P. vivax* in this antigenic locus. In the present study, using sequence data derived from *P. vivax* clinical isolates in patients from Ethiopia and genbank data, the nucleotide and amino acid sequence of the PvMSP3α block II were used to study evolution of the gene and potential consequences with respect to its use as a subunit vaccine candidate in light of LCRs.

## Results

### *PvMSP3α* gene subtypes and clonality of infection

Of the 50 dried blood spot (DBS) samples that were confirmed for *P.vivax* infection, the *MSP3α* gene was successfully amplified in 48 of the 50 samples. From the band size of the amplification products, compared to the molecular marker, 3 size variants were observed for *MSP3α* gene: type A (1 .9kb, 39 (82.9%), type B (1 .5kb, 6 (12.7%)) and type C (1 .1kb, 3 (4.2%)). A single multi-clonal sample with more than one band size was detected among the 48 samples. Excluding this sample, restriction digestion analysis using *Hha I* and *Alu I*, identified five additional multi-clonal samples; making an overall 12.5% (6/48) multiplicity of infection.

**Fig. 1:**
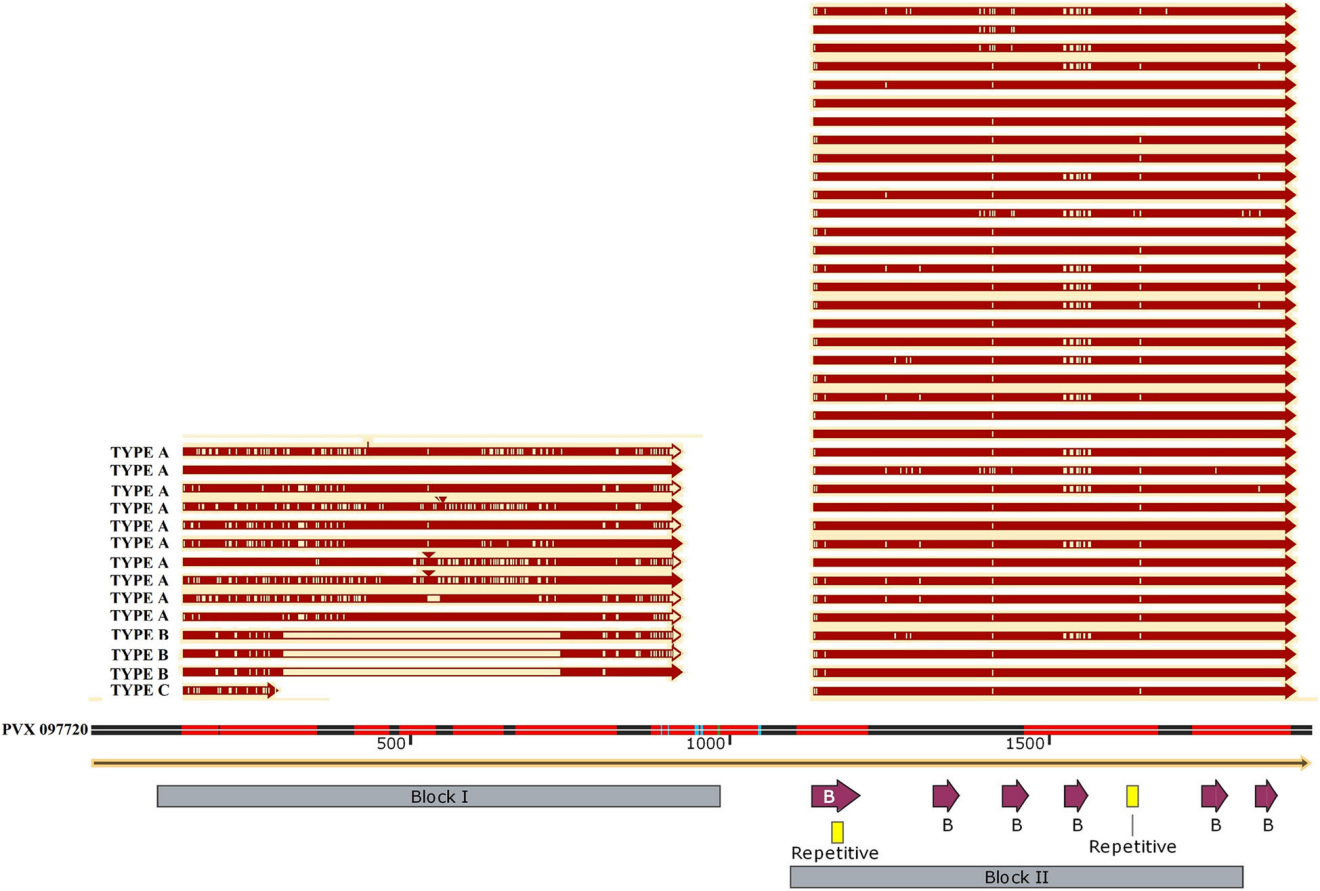
Multiple amino acid sequence alignment of the PvMSP3α block I and II with the reference sequence (PVX 097720). Shown in the left panel is the PvMSP3α block I with the three size variants (**a**, **b**, **c**) indicated from top to bottom, respectively. In the right panel, the relatively conserved *PvMSP3α* block II is indicated. The reference sequence (PVX_097720) was used to locate LCRs (red bars, imbedded within) and B cell epitopes (purple arrow, below it) in block II region

Of the total 15 samples that were sequenced for *PvMSP3α* entire region; one of the samples was not of sufficient quality. The three size variants observed during the PCR amplification are, type A that ranged from 1815 bp to 1925 bp, type B from 1407 bp to 1437 bp, and two representatives of type C‘s with a size of 1173 bp were observed. Type A variant sequences had several insertion and deletions, as evident in the size difference between the smallest and the largest sequences which amounted to 110 bp. Type B variants had an intact 281 bp at the start of the sequence followed by a deletion of 435 bp and ending with an intact block II compartment. The alignment of amino acid sequences (Fig. 1) deduced from the 14 full and additional 23 block II sequences from Ethiopian isolates and the reference sequence PVX_097720 showed that block II is relatively conserved compared to block I.

### The polymorphism and genetic diversity of *PvMSP3α* block II is limited to specific sites

**Fig. 2:**
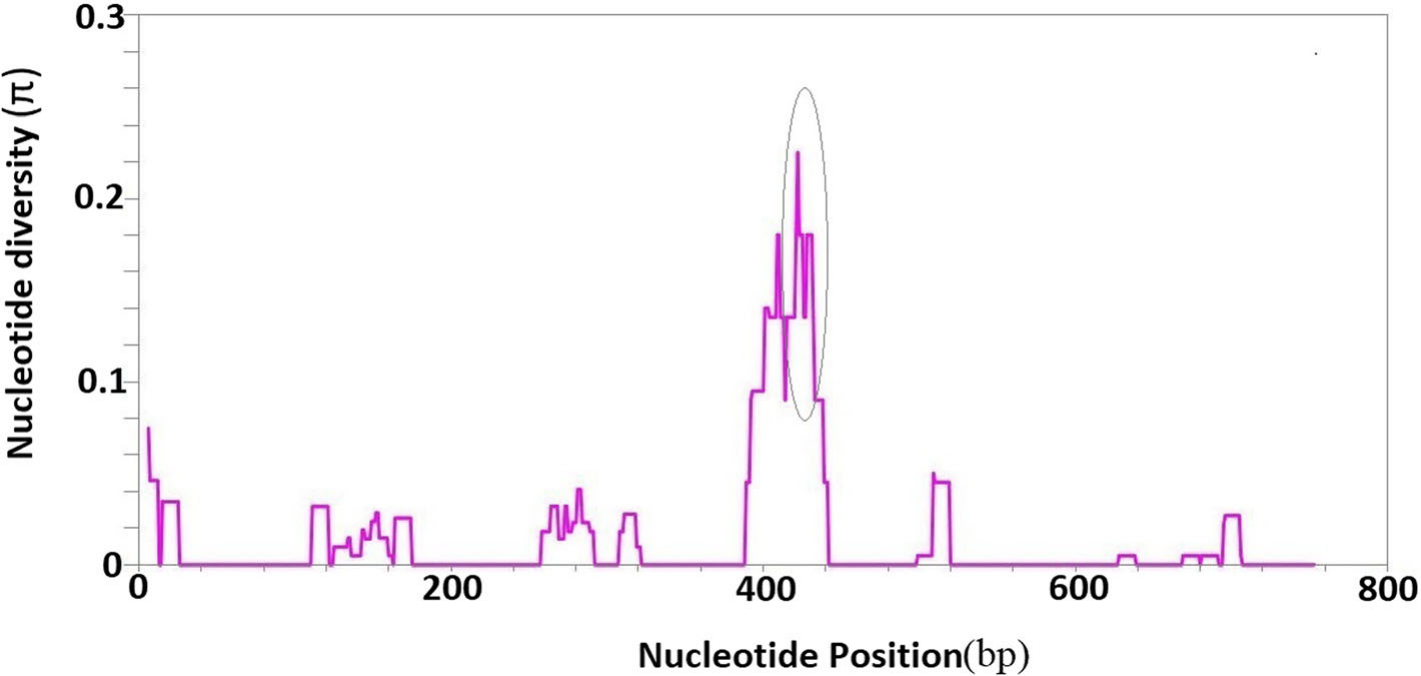
Sliding window plot analysis of nucleotide diversity in PvMSP3α block II in Ethiopian isolates. Histograms show the position (X-axis, bp refers to base pair) and diversity level (Y-axis) circle indicates peak diversity level attained

Of the total 40 Ethiopian isolates sequenced for *PvMSP3α* block II, three were deemed of low quality for analysis (1 sequenced for the full region as described above and 2 sequenced for block II region only). The alignment of the block II 758 bp (*n* = 37) identified 38 variable sites, of which 29 were parsimony informative (9 were singletons); 23 were non synonymous mutations. Rich nucleotide diversity (π = 0.013) with 20 haplotypes and a very high (0.953) haplotype diversity (Hd) was observed. However, the extent of diversity varied in different segments of the block, for instance superior values of diversity were attained between positions 404 bp and 436 bp of the alignment where a diversity as high as 0.22 was detected in this block (Fig. 2).

### Majority of the known B cell epitopes in *PvMSP3α* block II mapped in LCRs

Three LCRs interspersed within the 254 amino acid residues that define the block II region of the PvMSP3α were identified. The first LCR site spanned 29, the second 69 and the third 51 amino acid residues. In total, from the 254 examined residues, 158 (62.2%) were amino acids that encompassed the LCRs (Fig. 1).

The three LCRs shared a significant abundance of three amino acids; alanine, glutamic acid and lysine respectively. Over representation was particularly evident for alanine and lysine whose abundance spiked within LCR sites as compared to their overall composition in the block (Additional file 1: Table S1). For instance, the abundance of alanine within LCR2 and LCR3 was 39% as opposed to its overall composition (31%). Similarly, for lysine in contrast to its 16% overall abundance; within LCR1, LCR2 and LCR3, it had an abundance of 24, 20.29 and 21% respectively. Yet, the two high complexity regions (HCRs) sites adjacent to these LCR sites had lower abundance for alanine (24% & 15%) and lysine (9% & 15%).

**Fig. 3:**
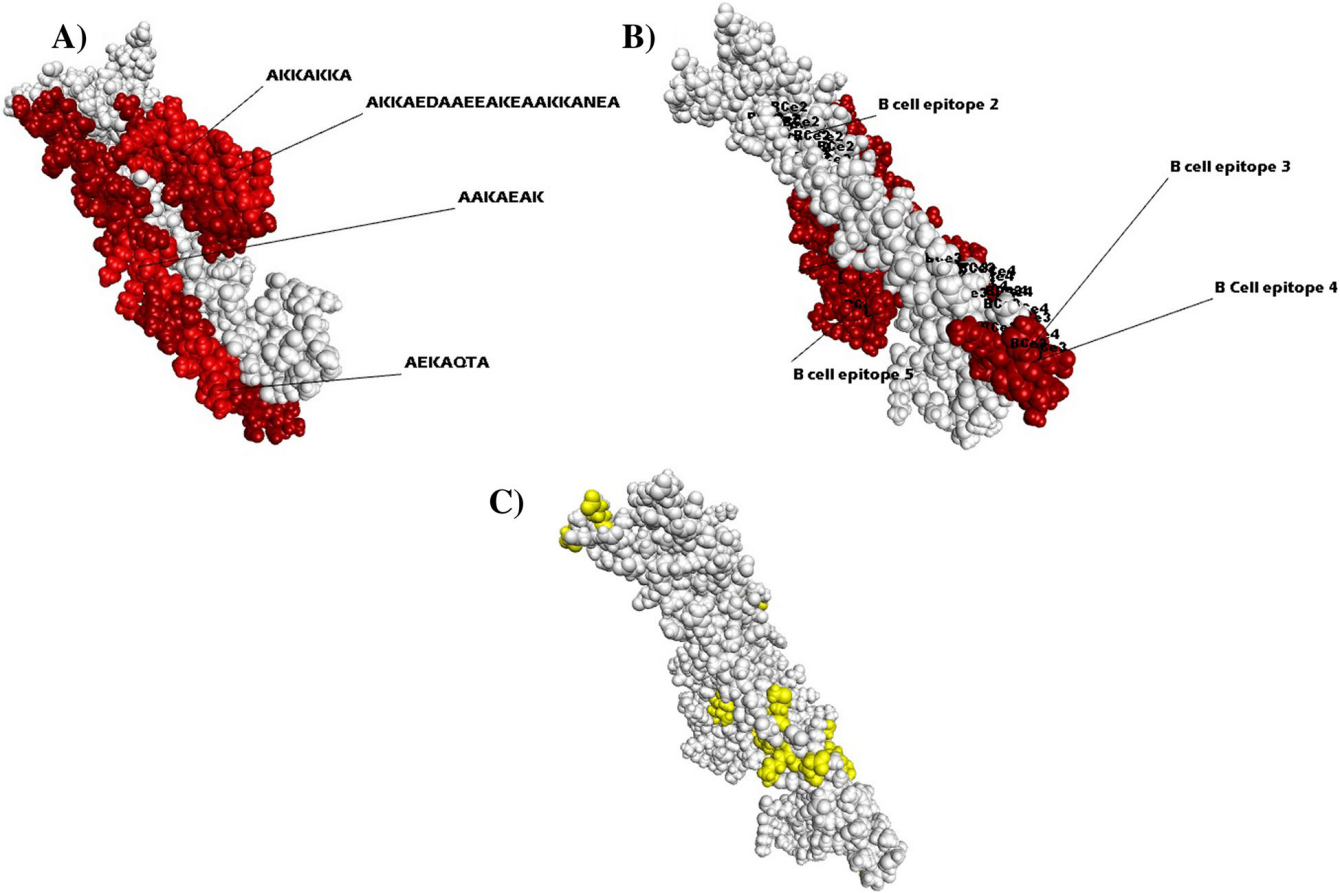
The predicted tertiary left handed coiled coil structure of *PvMSP3α* block II protein. Indicated in **a**) is the heptad repeat sequences (‘abcdefg’ configuration) and location within LCRs responsible for the tertiary structure, **b**) location of B cell epitopes (arrow) and LCR sites (red) in the tertiary structure, **c**) polymorphic sites in PvMSP3α block II (yellow) as investigated after aligning all global block II protein sequences

From the six known B cell epitopes within the block II region (Table 1), four were mapped within LCR sites, one each for LCR1 and LCR2 and two for LCR3 (Figs. 1 and 3). Curiously, also the two B cell epitopes found within the HCR sites also extended to the start of LCR2 (Fig. 3). Another interesting feature was the presence of repeats, particularly one located in a B cell epitope 1 embedded within LCR1 and the other located within LCR2, both exhibited the motif ‘AAAEEA’, while one is a B cell epitope, the other is not (Fig. 1). Two of the three aforementioned abundant amino acids, alanine and lysine, were also over represented in these six epitopes. Most notable was the abundance of lysine for LCR located B cell epitopes, ranging from 21.43 to 33.33% as compared to the two epitopes based on HCR site (7.14% &15.38%). Whereas alanine is abundant throughout all six (28.57–40%), and the abundance of glutamic acid was variable, from as high as 28.57 to 0%.

**Table 1:** B cell epitopes of the *PvMSP3* block II from the database https://www.iedb.org/: the location of epitopes, amino acid substitutions and the proportion of the variable amino acids were stated as observed by aligning 142 amino acid sequences from 18 populations

Epitope (IEDB)	Region Found	Position & Variable residues (substitutions)	% Proportion
1) NDATEAKKQAEKAKAAAEEAKTHGEK	LCR	1 K/N/H/E	68.6/28.7/1.1/1.1
		8 E/K	79.8/19.7
2) KAYAVEAHLAKTKN	HCR	Singletons Only	
3) DAANIAHQKWLKAT	HCR	Conserved Region	
4) KAQKEATAAKLKA	LCR	134 A/T	95.7/3.7
		136 T/K/N/A	54.8/43.6/0.5/0.5
		137 A/E	55.9/43.6
		139 N/T	54.8/44.7
		140 V/A	55.9/43.6
		141 V/A	55.9/43.6
		143 D/L/F	54.8/44.1
5) AEDAAEEAKEAAKK	LCR	212 A/V	94.1/5.9
		215 A/T/P	98.4/1.1
6) DKTIAAAKKAKKARE	LCR	234 K/N	97.3/2.7
		235 A/T	98.4/1.6
		236 I/M	98.9/1.1

### B cell epitopes and its flanking regions contained clustered polymorphic sites

In terms of amino acid polymorphism, a total of 37 amino acid mutations were observed in Ethiopian *P. vivax* isolates, of which 22 were singletons. A total of 15 sites were parsimony informative. Eight of these mutations were found in motif I and II (M/L E/K, K/E), (K/T, E/A, T/N. A/V, D/L) which are B cell epitopes in this block. Additionally, two amino acid changes were observed in the first 50 amino acid residues of the block (K/E, K/E, D/E), the first being within the B cell epitope region. The two remaining amino acid changes were found at residues 225 (D to N substitution) and 233 (K to N substitution), and the latter substitution was specific to Ethiopian *P. vivax* population. Whereas, the former substitution was only observed in two other isolates from Ethiopia, Brazil (Belem strain, AF093584.2) and Thailand (AY833015.1) but not observed in isolate sequences from other geographic regions. Interestingly the D to N substitution also lies in a previously characterized B cell epitope.

To map the global diversity of *PvMSP3α* block II amino acid sequence, 142 (37 Ethiopian, and 105 global) amino acid sequences derived from 12 populations were used for analysis. From this an overall 26 parsimony informative sites were observed in global isolates, 19 mapped to the 3 LCR sites and 14 of these were exclusive to the B cell epitopes within these regions. Only 7 polymorphic sites were identified within HCR sites, with 4 of these lying within motif I. The analysis for the conservation of the 6 epitopes in block II region revealed a peculiar variation. Four of the six epitopes located in the LCR sites had mutations, in contrast to the 2 epitopes found in HCR sites which remained conserved albeit low frequency singletons in B cell epitope 2 (Table 1). B cell epitope 4 specially had dimorphic alleles, with both forms showing equivalent proportion in terms of abundance. The other 3 epitopes (B cell epitope 1, 5, 6) also show amino acid substitution even though the frequency of the mutations was not as notable as that of B cell epitope 4.

To visualize the localization of the B cell epitopes and LCR sites, the tertiary structure of the PvMSP3α block II protein was modeled; the predicted protein formed α-helices with heptad repeats responsible for its tertiary left handed coiled-coil structure (Fig. 3 a).

In terms of B cell epitope localization, the six epitopes were mapped to the solvent exposed surface, further elucidating their role in immune mechanism (Fig. 3 b, Additional file 1: Table S1, Additional file 2: Table S2 and Additional file 3: Figure S1). Also, most of the polymorphic sites were clustered around or were in fact B cell epitope sites. It is also important to note that there was no bias regarding the location of polymorphisms as they seem to be randomly distributed to all solvent accessible faces of the tertiary structure (Fig. 3 c). As expected all polymorphisms in the 6 B cell epitopes sequences were in a manner that did not disrupt the tertiary structure of the protein, that is where hydrophobic amino acids were substituted by similar non polar side chains and hydrophilic amino acids substituted by similar amino acid chains.

### Phylogeny, signatures of balancing selection and recombination in the genes encoding PvMSP3α

**Fig. 4:**
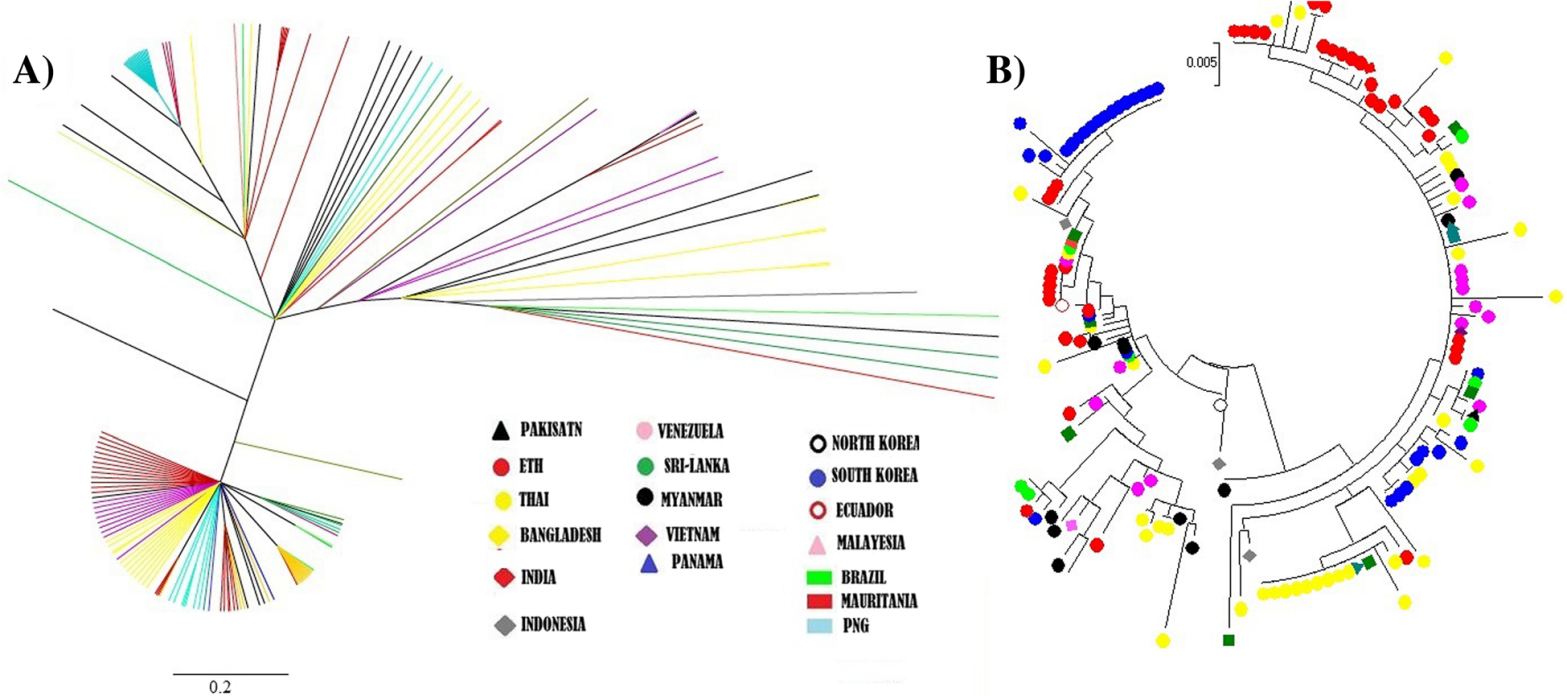
Phylogenetic trees based on PvMSP3α block II amino acid sequences from different geographical origins. Phylogenetic trees constructed using **a**) Bayesian inference (left) and **b**) maximum likelihood tree (right) based on block II sequences. The label of each sequence is color coded corresponding to the country of origin

Two phylogenetic trees were reconstructed using Bayesian inference, and maximum likelihood. In both cases, trees showed lack of obvious geographical structuring, for instance isolates from Ethiopia (red) were distributed in mini clusters and found in different parts of the cladogram, as was the case for most of the other isolates (Fig. 4).

For block II, apart from phylogenetic trees, evidence of population structuring was also minimal as observed using F_ST_ estimates for both local and global phylogenetic alignments, indicative of extensive gene flow. Instead, the phylogenetic tree clades were clustered based on the structural motif I & II dimorphic alleles. In addition, recombination was observed from isolates of different geographic origins and between different PCR size classes and throughout the phylogenetic tree generated by the RDP program. Similarly, a minimum of 6 recombination events (rm) were detected by Dnasp in the Ethiopian *P. vivax* population. These sites include those under balancing selection (motif I & motif II). Apart from this, RDP program identified 4 breakpoint events. These sites experiencing recombination were largely within LCR sites or sites immediately next to it. For instance motif II, which is under balancing selection and experiencing frequent intragenic recombination, lies within LCR2. Similarly, motif I which also experiences notable recombination lies adjacent to LCR2.

**Fig. 5:**
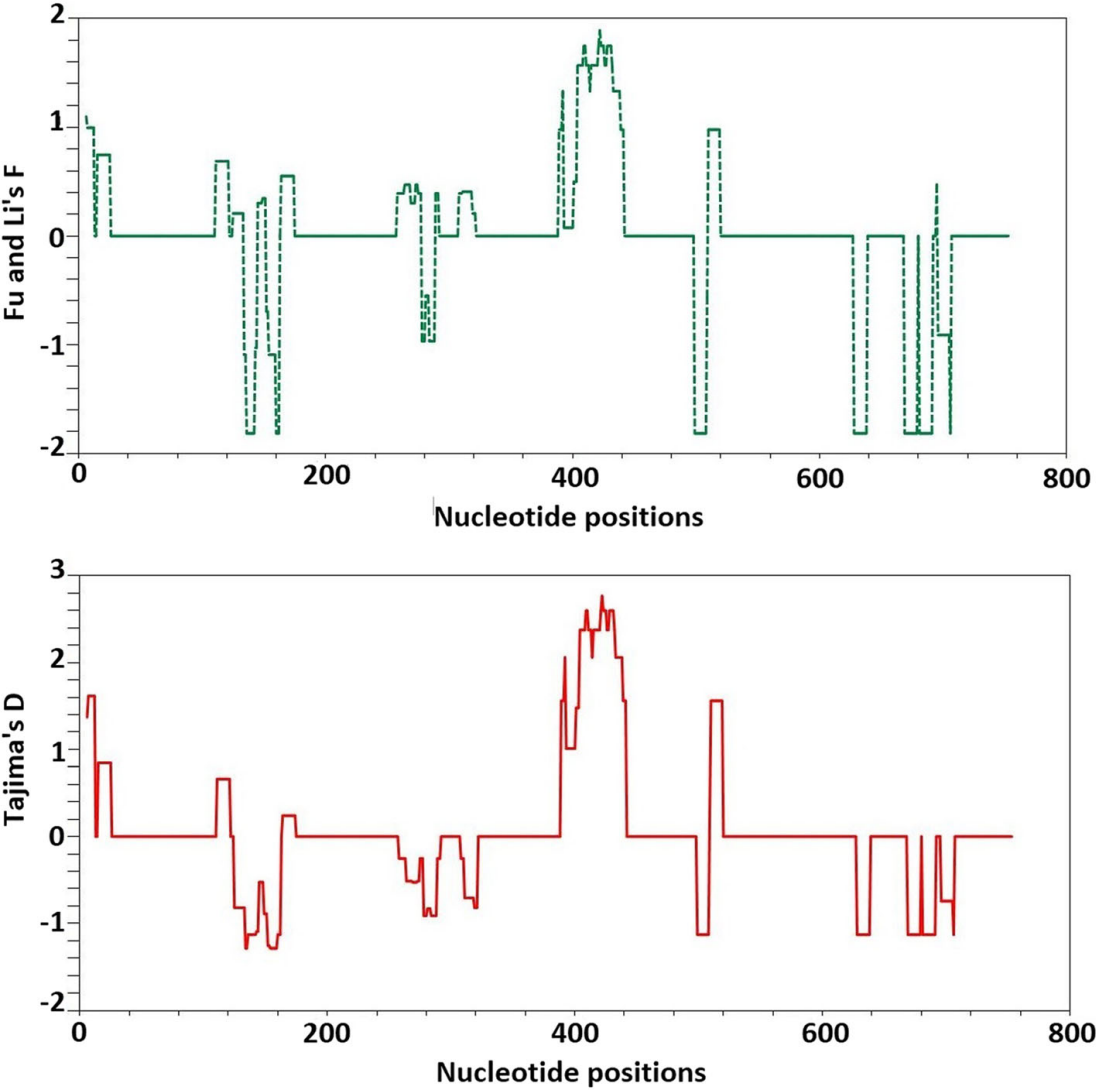
Sliding window plot analysis of signatures of selection on PvMSP3α block ii in Ethiopian sequences: Fu & li’s F (green) and Tajima’s D (Red) test values (X-axis) are indicated using a window size of 11 and step size of 1 bp (Y-axis)

To test for signs of balancing selection, the number of synonymous substitutions per synonymous site (dS) and non-synonymous substitutions per non-synonymous site (dN) was calculated. Accordingly the null hypothesis (H0:dN = dS) and the alternative purifying selection (dN < dS) were rejected at significant values of *P* = 0.049 and *P* = 0.026 respectively. Additionally, calculated frequency based tests of Tajima’s D and Fu and Li’s F tests were 0.702 (P>0.100) and 0.337 (P>0.100), respectively corroborating the aforementioned result. However, window plot analysis with window size of 11 bp and step length of 1 revealed significant value of Fu and Li’s F between the 387 bp and the 443 bp LCR region. Similarly the Tajima’s D statistic showed significant values between positions 399 bp–437 bp (Fig. 5). In this region Fu and Li’s F statistic was 1.762 (*P*>0.050), Tajima‘s D was 2.640 (*P*>0.050). The observations indicate that the small region with significant values of the parameters were under balancing selection. Comparative analysis of the 24 bp region encoding motif II revealed significant values of Fu and Li‘s F 2.291(*P* < 0.020) and Tajima’s D 3.230 (*P* < 0.001). Whereas the rest of the block minus the motif II sequence (693 bp) revealed significant values of − 5.323(*P* < 0.002) of Fu& Li’s F and − 2.566(*P* < 0.001) of Tajima‘s D. This region that encodes structural motif II, has dimorphic alleles TAANVVKD and KEATAAKL, indeed another region (motif I) with a dimorphic allele was also identified (MSELEK and LSKLEE) at a LCR adjacent part of the gene. Although its dimorphism was at a lesser extent in the Ethiopian *P. vivax* isolates. In contrast, the dimorphic alleles of motif II were equally prevalent (1:1) in the isolates.

**Fig. 6:**
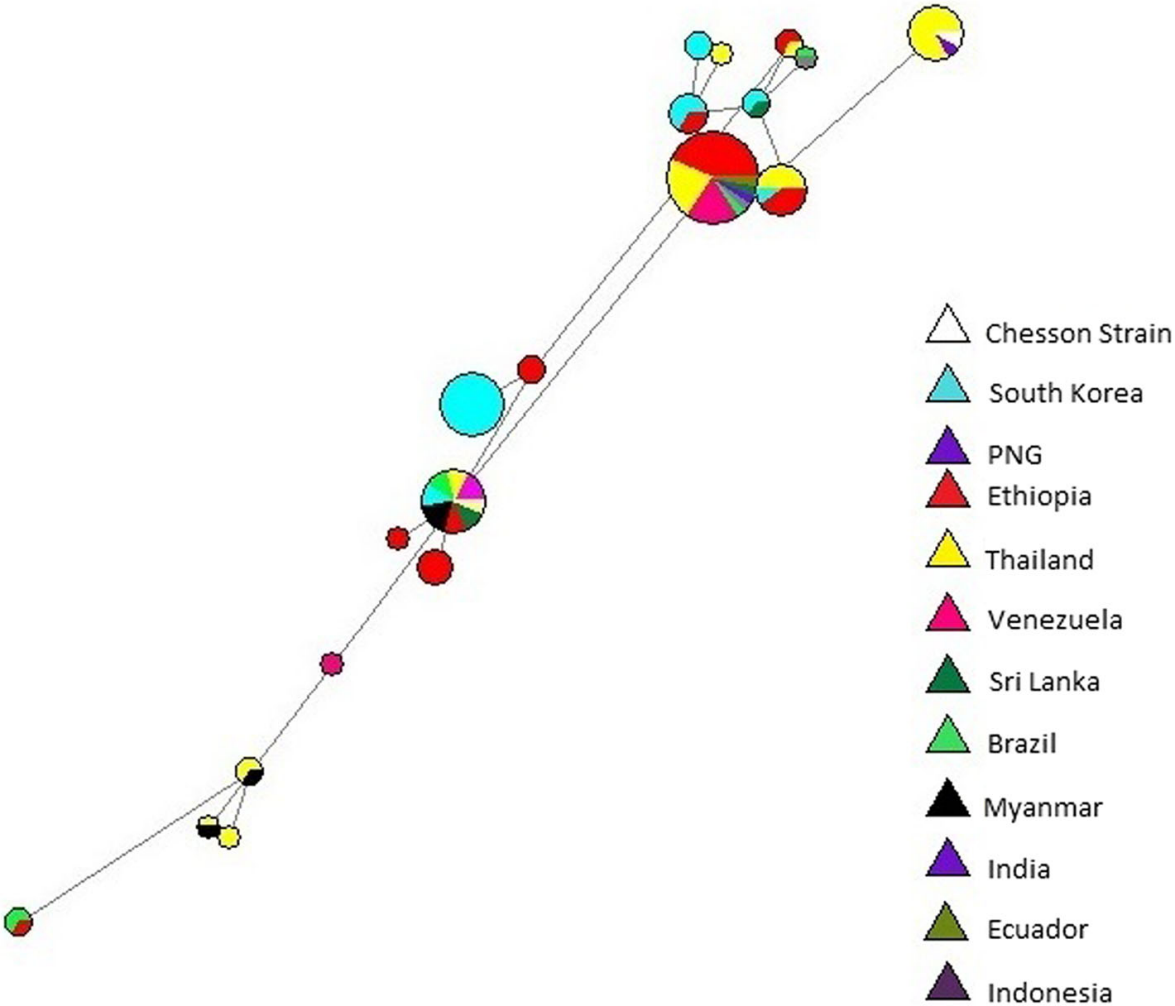
Haplotype Network for global *P.vivax* sequences that represent *PvMSP3α* block II region. Haplotypes were constructed from non-synonymous (*n* = 26) variations, each color represents a specific population (*n* = 12) as outlined by the legend. Node sizes represent frequencies and lines represent the mutational pathways

To further understand the impact of population structure or lack thereof on haplotypes, a network was drawn using the median joining algorithm (Fig. 6). To focus on the haplotypes that were frequent in the world and relevant to vaccine design, only non-synonymous variations that were seen in more than two isolates were used to construct the haplotypes. While the focus of the study was primarily the Ethiopian population, the haplotype network was derived from the 26 (non-synonymous) haplotypes of 12 populations. Accordingly 9 haplotypes with prominent frequency were commonly observed in the populations. Three haplotypes were observed in 51% of the isolates in this study, the most frequent haplotype included sequences from populations of Ethiopia, Sri Lanka, Papua New Guinea, Venezuela, Thailand, Myanmar, Vietnam and Panama. The second most frequent haplotype 1 also included sequences from the above populations as well as from Brazil, Ecuador, South Korea, India and Mauritania. The two haplotypes represent 13 of the 17 *P. vivax* endemic countries included in this study.

## Discussion

*PvMSP3α* block II domain is one segment of a vaccine candidate antigen that appears relatively conserved amongst global *P. vivax* isolates and has hence garnered more studies. This was also evident in the nucleotide diversity observed for the Ethiopian *P. vivax* population (0.014and haplotype diversity 0.953), which was comparable with other endemic countries; that ranged π 0.015(Brazil) to 0.023(India) while the global diversity stood at 0.019 [20]. Previous studies have also indicated that polymorphism was limited to specific sites of the block, this was also true for Ethiopian isolates; at and around motif I and motif II where π could be as high as π = 0.22 [19]. These peculiar patterns of genetic diversity are attributes of the functional constraints that are placed upon block II. Interestingly prior studies have shown that the alanine rich domain of block I and II are hot spots for recombination, but being particularly higher for block II than in block I where it could be 2 to 5 times higher [18, 21] . Indeed meiotic recombination is a common phenomenon in malaria antigens that is favored by multi-clonal infections, another recurring theme of *P. vivax* infections, which was higher in our study (12.8%) like previously reported [22] for multiplicity of infections.

Given the history of this block as a potential vaccine candidate that is also a hot spot for recombination, it was important to characterize intragenic recombination events and assess their contribution in evading the immune system. High frequency of recombination events combined with evidence of polymerase template slippage reported in prior studies, led to the hypothesis that LCRs might be present in this gene [18, 19]. This is largely because both mechanisms are associated with the origin of LCRs, but also because LCRs are common features in surface antigens of *Plasmodium* species [7, 18]. This was confirmed in this study by using the SEG algorithm. Particularly for block II, 3 LCRs were found interspersed within the domain. Furthermore, the association between recombination and LCRs was also confirmed. A minimum of 6 recombination events (rm) were detected in the Ethiopian *P. vivax* population. These sites experiencing recombination were largely within LCR sites or sites immediately next to it. For instance motif II, which is under balancing selection and experiencing frequent intragenic recombination, lies within LCR2. Similarly motif I, who also experiences notable recombination, lie adjacent to LCR2. These two motifs are interesting since both were particularly predicted to be B cell epitopes in previous in vitro and in silico studies (http://www.iedb.org/) [20]. Additionally since diversity in genes encoding antigens especially on the sporozoite and merozoite result from natural selection imposed by the immune system [23], we also performed tests of neutrality. Consequently test for neutrality was rejected at a significant value (*P* = 0.049, *P* < 0.050). Similarly the negative selection hypothesis was rejected at (*P* = 0.026,P < 0.05), suggesting that positive selection might be operating on block II. This finding is quite similar to the ones observed in a study by Rayner and colleagues in these antigen loci [19]. In positive selection, genetic variants favored by this pressure will either increase in frequency or be maintained, as has been the case for *P. falciparum* vaccine candidate antigens such as MSP-1, MSP-2, TRAP and AMA-1 [24]. Although in the current study, positive values were observed for both Tajima‘s D and Fu and Li‘s F across the length of the block II, highly significant values were observed only in one specific region, the LCR region encoding motif II. Further comparative analysis of the structural motif II (24 bp) and rest of the block (692 bp) also revealed a highly significant positive value for both tests in the structural motif II. In contrast, the rest of the block had significant negative values. This would indicate that, while positive selection is operating on the entire block thus reducing diversity, the small region encoding motif II is under balancing selection (Immune selection). As further evidence on the effects of balancing selection, genetic differentiation estimates for selected population sequences revealed low F_ST_ estimates (data not shown). Moreover, F_ST_ values between Ethiopian and Brazilian (F_ST_ = − 0.02), Ethiopian and Sri Lankan MSP3α block II isolates (F_ST_ = 0.025) are lower than values attained using single nucleotide polymorphism markers (SNP) for both pairs (Ethiopia and Sri lanka F_ST_ = 0.21; Ethiopia and Brazil F_ST_ = 0.31) [25]. However, this data should be interpreted with caution because of the small sample size in the study.

The lack of geographical structuring was further supported by phylogenetic tress that were constructed using global sequences from 12 populations using block II domain. These results are consistent with those of other studies and suggest that phylogenetic inference alone may not be sufficient in vaccine design using this protein [17, 18, 21, 26]. From the constructed haplotypes of block II, 3 of the haplotypes were shared in 51% of the sequences included in the current study. The results were more positive than the vaccine candidate antigen PvAMA-1, where only 15% of the haplotypes were shared among studied global isolates [27]. However it is also important to bear in mind that due to the limited number of samples, results should be interpreted with caution. Apart from allelic diversity and given that this is a surface protein other immune evading mechanism are also likely, as reviewed in Ferreira et al., 2004 [13]. One possibility is the generation of LCRs, which have been associated with both protein-protein interaction as well as immune evasion. LCRs were identified with biased composition sharing a significant abundance of 3 amino acids; alanine, glutamic acid and lysine. Curiously, a repeat of ‘AAAEEA’, was found embedded within LCRs; one serving as a B cell epitope while the other is not. This might indicate immune mimicry affecting antibody affinity maturation and hence lowering the efficiency of response to critical epitopes [12].

The PvMSP3α protein was predicted to form α-helix structure with heptad repeats responsible for its tertiary left handed coiled-coil structure as described earlier [16]. Interestingly, most of the known B cell epitopes were found within this LCR sites; in addition LCRs dominate solvent exposed side of the protein in sharp contrast to their high alanine (hydrophobic) content. This might be counterintuitive; however alanine is a small amino acid that is not particularly homophobic and hence tolerated on protein surfaces. Additionally the amphipathic nature of the heptad repeats also supports this configuration;-Moreover, the high alanine content in LCRs is equivalent to the combined abundance of the polar amino acids lysine and glutamic acid. In terms of B cell epitope localization, all 6 analyzed epitopes were solvent exposed. Furthermore most of the polymorphic sites were clustered around or were in fact B cell epitope sites elucidating their role in immune mechanism. It is also important to note that there was no bias regarding the location of polymorphisms as they seem to be randomly distributed to all solvent accessible faces of the tertiary structure. As expected all polymorphisms in the 6 B cell epitopes sequences were in a manner that didn’t disrupt the tertiary structure of the protein*,* that is where hydrophobic amino acids would be substituted by similar non polar R groups and hydrophilic amino acids would be substituted by similar R groups. However, even in such like-for-like substitutions the observed alanine substitution warrant further investigation, since alanine mutations have been shown to decrease immune-reactivity in previous studies using Apical membrane antigen 1 of *P. falciparum* [28]. The reason behind this reduced immunogenicity imparted by alanine residues is thought to be the result of its rigid nature resulting in loss of epitope flexibility and hence reduction in antibody binding [28]. As such it is also critical to assess substitution polymorphisms in-terms of their structural impact. Hence the peculiar variation of motif II, a B cell epitope with alternate dimorphic alleles (TAANVVKD/KEATAAKL represents a clinically relevant finding not only due to its dimorphism but also because of structural consequences of this substitutions. To further complicate matters, LCRs are also associated with phenotypic plasticity, and given the proteins dependence in block II domain for peripheral association with other proteins, in addition to the surface proteins involvement in merozoite invasion; it is likely that antigenic diversity mechanisms described for invasion ligands Erythrocyte binding antigen (EBA) and reticulocyte binding-like homologous (PfRH) protein also apply here [29–31].

## Conclusion

The abundance of LCRs in *PvMSP3a* block II has contributed/influenced the tertiary structure of the protein. Furthermore there is an enhanced site preference of balancing selection, recombination and repeat motifs to map on LCRs. The predicted B cell epitopes were also in or adjacent to the LCRs which indicate the strong phenotypic plasticity associated with this domain. Thus our results are indicative of LCRs contribution toward immune escape mechanisms and lack of geographic clustering in *PvMSP3α* block II.

## Materials and methods

### Study area, sample collection, and ethics statement

The study was conducted between 2012 and 2013 at Shewa Robit town health center (9° 59′ 40.6″N and 39° 53′ 48.9″E) and five health facilities in Shala district; Aje (7° 17′ 34.2″N and 38°21′ 46.3″E), Bure (7°15′ 7.25°N and 38° 29′ 38.4833°E), Haposto (6.7377904 and 38.3691932), Ilala (8°55′ 28.27″N,39° 50′35.90″E) and Melka Oda hospital (l7°13′ 7.2167°N 38°29′ 38.4833°E). Finger prick blood samples were collected to prepare thin and thick blood films for parasite identification using microscopy and dried blood spots (DBS) on Whatmann 3MM filter papers.

### Species identification, amplification of the *PvMSP3α* gene and restriction digestion

Genomic DNA was extracted by Chelex-Saponin dual extraction method [32] from 6 mm diameter DBS punches. Nested polymerase chain reactions (nPCR) that targeted the 18S small subunit rRNA gene were run to confirm species of malaria infections [33, 34].

The block I and II of the *PvMSP3α* genes were amplified using primers and PCR conditions described before [35]. Nested PCR products were then digested using two endonucleases, *Hha I* (Promega, USA) and *Alu I* (Sigma Aldrich, USA*)* using reaction conditions described before [36]. The PCR products were visualized on 0.8% agarose gel (AGCT ltd, USA) and digested products were run using 1.8% agarose and 1 kb plus molecular ladder (Invitrogen, USA) alongside for estimating sizes of products.

### Sequencing and population genetic analysis

Polyclonal infections were first discerned from mono infections by using PCR-RFLP procedure as reported by Bruce and colleagues [35]. PvMSP3α nested PCR products were then purified using the QIAquick PCR purification kit (QIAGEN, Germany,) and the template was sequenced using outer and internal primers, as described before [36]. Consequently, samples targeting the entire block (I and II) and additional 25 samples targeting block II region only were sequenced twice, both in forward and reverse direction, using the Big Dye terminator sequencing kit and the ABI Prism 310 Genetic analyzer (Applied Bio-systems, base clear, The Netherlands).

Sequences were first inspected visually to ensure correct base calls of the chromatogram data using Chromas (version 2.6.4, Technelysium LTD). The low quality regions were trimmed, assembled, and individually aligned using ClustalW [37] to two MSP3α reference sequences; the Belem (AF093854) and Salvador strain (PVX_097720) using SeqMan Pro 14 (Lasergene 14 software, DNASTAR Inc.).

To study the phylogenetic relationships among block I and II sequences both aligned nucleotide and deduced amino acid sequences were used. Trees were constructed using the maximum likelihood with Tamura and Nei model of nucleotide substitution for nucleotide alignments [38] and the Jones Taylor Thronton model of amino acid substitution method for amino acid alignments [39] with 1000 bootstrap replicate support for both using the MEGA7 software (Version 7.0.21). Furthermore, phylogenetic trees were also constructed for both blocks using Bayesian inference as implemented by Mr. Bayes (Version 3.2.6) using a general time reversible gamma evolutionarily invariable (GTR + G + I) model. Results were acquired after using 39 × 10^6^ MCMC steps and convergence was reached, subsequently 50% of the samples were discarded as burn-in.

The Dnasp Software (Version6.10.04, Universitat de Barcelona) was used to explore sequence diversity; such as, the number of polymorphic sites (S), within population and overall nucleotide diversity (π), number of haplotypes (H) and haplotype diversity (Hd) [40]. To determine genetic differentiation, Wrights Fixation index F_ST_ was tested through 1000 random permutations [41]. To assess departure from neutrality and examine if regions were under selection, the number of synonymous substitutions per synonymous site (dS) and non-synonymous substitutions per non-synonymous site (dN) was calculated using the modified Nei Gojobori method [42]. The null hypothesis of neutrality (H0: dN = dS), and alternative hypothesis of positive selection (dN > dS) and purifying selection (dN < dS) were tested using a two tailed Z test for neutrality and one tailed Z test for either of the alternative hypothesis. Standard errors were computed through 1000 bootstrap replicates. Furthermore, Tajima‘s D [43] Fu and Li‘s F [44] were applied using a sliding window approach to investigate signatures of balancing selection. Finally Dnasp was used for haplotype construction. NETWORK (Version 5.0.0.1, Fluxus Technology Ltd) was used to create and visualize haplotype networks by applying the median Joining algorithm. To detect recombination signals RDP4 program was used [45]. In order to analyze the dataset generated in this study to a global context, 126 MSP3α sequences from 17 *P. vivax* endemic countries were retrieved from genebank (http://www.ncbi.nlm.gov/genbank).

Low Complexity Regions (LCRs) of the PvMSP3α, Salvador strain (PVX_097720) and the Belem reference sequence (AF093584) was detected using the SEG algorithm, http://mobidb.bio.unipd.it/. Tandem repeats were detected using Ugene (v 1.29) and XSTREAM, http://jimcooperlab.mcdb.ucsb.edu/xstream/. LCRs were determined using the SEG algorithm as specified before [46, 47]. Further, to analyze variable site across B cell epitopes, experimentally identified epitopes were extracted from the Web server (http:/https://www.iedb.org/).

### Structural modeling of PvMSP3α

Since there are no homologous proteins specified to date for neither the PvMSP3α nor reliable templates to perform homology modeling, an Ab initio method was used to determine the tertiary structure. Accordingly the ROBETTA server, *http://robetta.bakerlab.org/**,* was used to construct 10,000 decoys for PvMSP3α Salvador strain (PVX_097720) filtered and clustered based on the root-mean-square deviation of atomic positions (RMSD), subsequently the top 5 models were chosen based on lowest probability density function (PDF) [48]. The models were then further evaluated in the ResProx (http://www.resprox.ca/) and Vadar (http://vadar.wishartlab.com/index.html) servers [49, 50]. Finally, they were annotated using the Discovery studio visualizer version 17.2 (Accelrys, San Diego, CA). The Ab Initio methods of the I-Tasser *(**https://zhanglab.ccmb.med.umich.edu**)* and QUARK as well as the University of reading server IntFOLD3 (*http://www.reading.ac.uk/bioinf/IntFOLD/*) were also used to compare each of their models [51, 52].

## Additional files

Additional file 1: Amino acid compostion(percent) within Plasmodium vivax merozoite surface protein 3α (PvMSP3α) block II. (DOCX 16 kb)

Additional file 2: Accession numbers of Plasmodium vivax merozoite surface protein 3α (PvMSP3α) sequences retrieved from GenBank. (DOCX 14 kb)

Additional file 3: Ramachandran plot of predicted tertiary structure. (PNG 19 kb)
