# Supplementary material for: Effect of low complexity regions within the PvMSP3α block II on the tertiary structure of the protein and implications to immune escape mechanisms
Source: BMC Struct Biol. 2019 Mar 27;19:6. doi: 10.1186/s12900-019-0104-0 (PMC6437935; doi:10.1186/s12900-019-0104-0)
Supplement: Supplementary file 1 — Amino acid compostion(percent) within Plasmodium vivax merozoite surface protein 3α (PvMSP3α) block II. (DOCX 16 kb) [file 12900_2019_104_MOESM1_ESM.docx]

| Amino acid | Overall | **Low complexity region** | | | **High complexity region** | |
| --- | --- | --- | --- | --- | --- | --- |
|  |  | **LCR1** | **LCR2** | **LCR3** | **HCR1** | **HCR2** |
| Alanine (A) | 31.00 | 31.03 | 39.13 | 39.22 | 24.39 | 15.79 |
| Arginine (R) | 1.97 | 0.00 | 1.45 | 3.92 | 2.44 | 0.00 |
| Aspargine (N) | 4.33 | 0.00 | 1.45 | 5.88 | 6.10 | 10.53 |
| Aspartic acid (D) | 3.94 | 3.45 | 1.45 | 5.88 | 6.10 | 0.00 |
| Glutamic acid (E) | 16.93 | 24.14 | 14.49 | 15.69 | 17.07 | 21.05 |
| Glutamine (Q) | 3.15 | 3.45 | 4.35 | 0.00 | 3.66 | 5.26 |
| Glycine (G) | 1.97 | 3.45 | 1.45 | 0.00 | 1.22 | 5.26 |
| Histidine (H) | 1.57 | 3.45 | 0.00 | 0.00 | 3.66 | 0.00 |
| Isoleucine (I) | 1.57 | 0.00 | 1.45 | 1.96 | 2.44 | 0.00 |
| Leucine (L) | 2.36 | 0.00 | 0.00 | 0.00 | 4.88 | 0.00 |
| Lysine (K) | 16.93 | 24.14 | 20.29 | 21.57 | 9.76 | 15.79 |
| Methionine (M) | 0.39 | 0.00 | 0.00 | 0.00 | 1.22 | 0.00 |
| Proline (P) | 0.39 | 0.00 | 0.00 | 0.00 | 0.00 | 5.26 |
| Serine (S) | 3.15 | 0.00 | 0.00 | 1.96 | 7.32 | 5.26 |
| Threonine (T) | 4.72 | 6.90 | 5.80 | 1.96 | 3.66 | 10.53 |
| Tryptophan (W | 0.39 | 0.00 | 0.00 | 0.00 | 1.22 | 0.00 |
| Tyrosine (Y) | 0.79 | 0.00 | 0.00 | 0.00 | 1.22 | 0.00 |
| Valine (V) | 4.33 | 0.00 | 8.70 | 0.00 | 3.66 | 5.26 |
