# Supplementary material for: Effect of low complexity regions within the PvMSP3α block II on the tertiary structure of the protein and implications to immune escape mechanisms
Source: BMC Struct Biol. 2019 Mar 27;19:6. doi: 10.1186/s12900-019-0104-0 (PMC6437935; doi:10.1186/s12900-019-0104-0)
Supplement: Supplementary file 2 — Accession numbers of Plasmodium vivax merozoite surface protein 3α (PvMSP3α) sequences retrieved from GenBank. (DOCX 14 kb) [file 12900_2019_104_MOESM2_ESM.docx]

| **Accession number** | **Isolate Origin** | **Accession number** | **Isolate Origin** | **Accession number** | **Isolate Origin** |
| --- | --- | --- | --- | --- | --- |
| KC935447.1 | Panama | AF491951.1 | Bangladesh | EF204163.1 | South Korea |
| KC935447.1 | Vietnam | EF204162.1 | South Korea | AJ864967.1 | Venezuela |
| KC935445.1 | Mauritania | EU430600.1 | Myanmar | AJ864944.1 | Venezuela |
| AF491958.1 | North Korea | AF491954.1 | Sri-lanka | AJ864942.1 | Venezuela |
| AY266090.1 | Pakistan | AF491961.1 | Sri-lanka | AJ864941.1 | Venezuela |
| AF491959.1 | Papua new Guinea | GU175269.1 | Sri-lanka | KC935431.1 | Venezuela |
| KC935444.1 | Papua new Guinea | GU175270.1 | Sri-lanka | KC935430.1 | Venezuela |
| AY266089.1 | Malaysia | GU175271.1 | Sri-lanka | KC935429.1 | Venezuela |
| AY118174.1 | Indonesia | GU175272.1 | Sri-lanka | KC935428.1 | Venezuela |
| AY266091.1 | Indonesia | GU175277.1 | Sri-lanka | KC935427.1 | Venezuela |
| KC935441.1 | Indonesia | GU175279.1 | Sri-lanka | KC935426.1 | Venezuela |
| AF491957.1 | India | GU175285.1 | Sri-lanka | KC935425.1 | Venezuela |
| HQ328854.1 | India | EU430576.1 | Myanmar | KC935424.1 | Venezuela |
| HQ328855.1 | India | EU430577.1 | Myanmar | KC935423.1 | Venezuela |
| HQ328853.1 | India | EU430578.1 | Myanmar | KC935422.1 | Venezuela |
| KC935446.1 | India | EU430579.1 | Myanmar | KR905529.1 | Thailand |
| AF491952.1 | Ecuador | EU430580.1 | Myanmar | KR905528.1 | Thailand |
| AF491953.1 | Ecuador | EU430581.1 | Myanmar | KR905527.1 | Thailand |
| AF491945.1 | Brazil | EU430582.1 | Myanmar | KR905526.1 | Thailand |
| AF491946.1 | Brazil | EU430583.1 | Myanmar | KR905525.1 | Thailand |
| AF491947.1 | Brazil | EU430584.1 | Myanmar | KR905524.1 | Thailand |
| AF491948.1 | Brazil | EU430585.1 | Myanmar | KR905523.1 | Thailand |
| AF491949.1 | Brazil | EU430586.1 | Myanmar | KR905522.1 | Thailand |
| JQ317283.1 | South Korea | EF204155.1 | South Korea | KR905521.1 | Thailand |
| JQ317284.1 | South Korea | EF204156.1 | South Korea | KR905520.1 | Thailand |
| JQ317285.1 | South Korea | EF204157.1 | South Korea | KR905519.1 | Thailand |
| JQ317286.1 | South Korea | EF204158.1 | South Korea | KR905518.1 | Thailand |
| JQ317287.1 | South Korea | EF204159.1 | South Korea | KR905517.1 | Thailand |
| JQ317288.1 | South Korea | EF204161.1 | South Korea | KR905516.1 | Thailand |
| JQ317289.1 | South Korea | AF491962.1 | Thailand | KR905515.1 | Thailand |
| AY266087.1 | South Korea | AY833010.1 | Thailand | KR905514.1 | Thailand |
| EF204144.1 | South Korea | AY833011.1 | Thailand | KR905513.1 | Thailand |
| EF204145.1 | South Korea | AY833012.1 | Thailand | KR905512.1 | Thailand |
| EF204146.1 | South Korea | AY833013.1 | Thailand | KC935442.1 | Thailand |
| EF204147.1 | South Korea | AY833014.1 | Thailand | AY833026.1 | Thailand |
| EF204148.1 | South Korea | AY833015.1 | Thailand | AY833025.1 | Thailand |
| EF204149.1 | South Korea | AY833016.1 | Thailand | AY833024.1 | Thailand |
| EF204150.1 | South Korea | AY833017.1 | Thailand | AY833023.1 | Thailand |
| EF204151.1 | South Korea | AY833018.1 | Thailand | AY833022.1 | Thailand |
| EF204152.1 | South Korea | AY833019.1 | Thailand | AF491956.1 | Brazil |
| EF204153.1 | South Korea | AY833020.1 | Thailand | KR905530.1 | Thailand |
| EF204154.1 | South Korea | AY833021.1 | Thailand |  |  |
