# Supplementary figures and images for: Effect of low complexity regions within the PvMSP3α block II on the tertiary structure of the protein and implications to immune escape mechanisms
Source: BMC Struct Biol. 2019 Mar 27;19:6. doi: 10.1186/s12900-019-0104-0 (PMC6437935; doi:10.1186/s12900-019-0104-0)

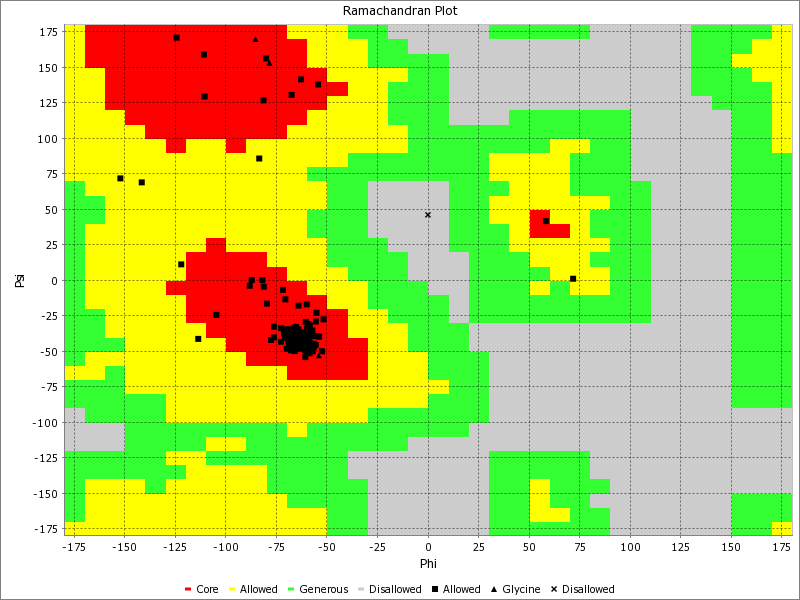

Supplement: Supplementary file 3 — Ramachandran plot of predicted tertiary structure. (PNG 19 kb) [file 12900_2019_104_MOESM3_ESM.png]
